# Insights into the identification of antimicrobial peptides: A multidisciplinary observation

**DOI:** 10.1002/imo2.41

**Published:** 2024-11-05

**Authors:** Sizhe Chen, Qi Su

**Affiliations:** ^1^ Microbiota I‐Center (MagIC) Hong Kong SAR China; ^2^ Department of Medicine and Therapeutics, Faculty of Medicine The Chinese University of Hong Kong, Hong Kong Special Administrative Region Hong Kong China

## Abstract

Antimicrobial peptides (AMPs) exemplify the principle of “Life finds a way” by adapting to diverse environmental niches. These peptides exhibit remarkable specificity, targeting pathogens unique to their respective habitats. Understanding the ecological context of AMPs not only clarifies how these peptides selectively target pathogens while remaining nontoxic to hosts, but also underscores the potential of computational approaches in AMP discovery and design. By integrating biological omics data with computational modeling, researchers can develop novel AMPs tailored to combat specific microbial threats, paving the way for innovative therapeutic solutions in an ever‐changing microbial landscape.

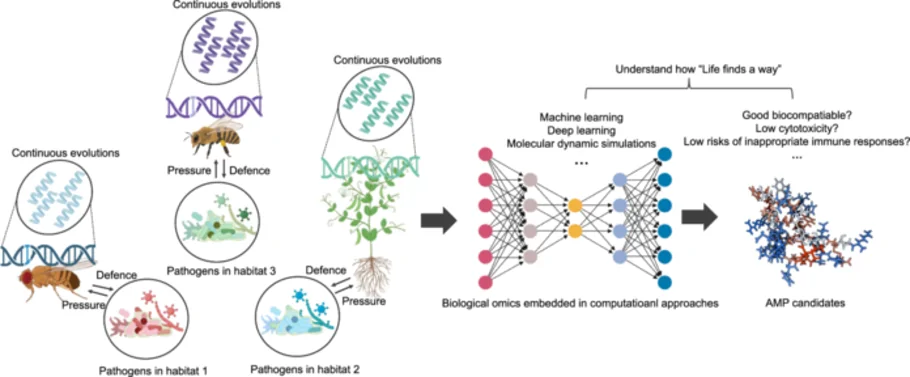

In the movie “Jurassic Park,” the line “Life finds a way” articulated by Ian Malcolm, encapsulates a fundamental theme regarding the resilience of biological life. This tenacity is particularly relevant in the context of antimicrobial peptides (AMPs), which are evolutionary gifts across nearly all multicellular organisms for combating natural enemies while showing biocompatibility to their own hosts [1]. Generally, AMPs are cationic and amphipathic macromolecules, tearing microbial membranes via detergent‐like manners without targeting particular biological targets [2]. Parallel to the event of prokaryotic origin 2.6 billion years ago, AMPs genes have shown continuous mutations with rates much faster than 5′‐UTR and 3′‐UTR regions, reflecting host adaptability to particular pathogens in the environment [3]. For example, anurans‐encoded AMPs have shown geographic‐specific profiles, with continuous mutations since the event of fragmentation of the Gondwana super‐continent [3]. The AMPs produced from gut symbionts of *Apis mellifera* are vital for combating *Melissococcus plutonius*, while showing no cytotoxicity to *A. mellifera* [4]. In *Homo sapiens*, this opinion is well‐supported by ancient host immune defense peptides called defensins, having millions of years of evolutionary history [5]. However, for an extended period, the evolutionary significance of AMPs has been overlooked, particularly in the context of their perfect host biocompatibilities and low cytotoxicity.

In the last decade, the semi‐rational design or discovery of AMPs has benefited from computational strategies, especially the artificial intelligence (AI) deep learning and machine learning algorithms, with reasonable overall accuracy and precision [6]. These advanced technologies rely on huge quantities of relevant data collected from experiments (Figure S1 and Table S1), and have achieved reasonable overall accuracy and precision in tasks of predicting and designing AMPs. The representative computational tools in recent years have also been summarized in Table S2. While computational strategies can predict or design AMPs with good minimum inhibition concentration values or hemolysis indicators [6], these peptides are not exactly the same as those naturally evolved AMPs. One of the major concerns with artificially designed peptides lies in their potential to elicit inappropriate immune responses or to exhibit low biocompatibility. The complexities of the immune system and the biocompatibility of synthetic peptides often exceed the predictive capabilities of the current computational technologies. In contrast, AMPs derived from natural organisms have undergone millions of years of evolution, fine‐tuning their properties to minimize off‐target cytotoxicity and inappropriate immune activation while maximizing their antimicrobial efficacy against specific pathogens. This evolutionary process has equipped natural AMPs with a refined selectivity that is often lacking in their synthetic counterparts [1, 7]. With growing challenges posed by antibiotic resistance, integrating insights from natural selection with computational strategies will be essential for developing effective and safe therapeutic agents. There is no doubt that AMPs exemplify how “Life finds a way,” as they represent the remarkable adaptability and resilience of living organisms.

## 1 ANTIMICROBIAL PEPTIDES—NATURAL DEFENSE WEAPONS WITH EVOLUTIONARY WISDOM

AMPs indeed represent a fascinating intersection of evolutionary biology and potential therapeutic applications, actively involved in modulating immune responses and maintaining homeostasis within various ecosystems. However, for a long period, AMPs were thought as broad‐spectrum or nondiscriminative antimicrobial agents. This viewpoint has been challenged in the last decade. In fact, AMPs are not merely macromolecules with antimicrobial properties; rather, they are sophisticated components finely tuned by evolution to exert selective pressure on pathogens.

A noteworthy type of AMPs is defensins, which are widely present in eukaryotic organisms. The similarities among defensins isolated from plants, insects, invertebrates, and fungi, indicate a common evolutionary origin believed to date back at least 114 million years ago [8]. In modern *H. sapiens*, defensins are potent AMPs with good host biocompatibility, showing delicate immune mediation activities and limited off‐target cytotoxicity [9]. Surprisingly, AMPs encoded by distinct anurans have been demonstrated to show geographically driven profiles in their sequence patterns. In detail, molecular phylograms revealed that the AMP genes among hylids and ranids dispersed across regions of South America, Australia, Asia, North America, and Europe shared common ancestral AMP origins and diversified by gene duplications and mutations in later generations. However, such diversifications in AMP genes are shockingly associated with particular geographical locations in later generations, suggesting potential links to pressures from certain environmental microorganisms. While once considered superfluous, the diversifications of AMP‐encoding genes are thought to be specifically “designed” for combating particular environmental pathogens. The *Drosophila* species coliving with particular microorganisms associatively harbor certain types of AMPs polymorphisms and show divergent targeting sensitivities [10]. In detail, Diptericin A and Diptericin B have been known to selectively against the pathogenic *Providencia* and *Acetobacter* species, respectively. *Drosophila* species living with *Acetobacter* consistently relied on the Diptericin B gene, while those living with a lack of *Acetobacter* microorganisms showed depletions of the Diptericin B gene. In an ecology lacking both pathogenic *Providencia* and *Acetobacter* microorganisms, *Drosophila* species even lost both Diptericin A and Diptericin B genes. Similarly, polymorphisms in genes encoding AMP Calprotectin (Gly/Glu) have rendered cats with different sensitivities to fungal infections [11]. A novel AMP called epifadin has been recently isolated from human nasal symbiotic *Staphylococcus epidermidis*; due to the short existing half‐time of epifadin, the collateral damage to human hosts is restricted [12]. AMP Melittin secreted from the venom of honeybee *A. mellifera* shows fatal cytotoxicity to mammalian cells and causes pain sensation in the human body, while not reveals threats to honeybee *A. mellifera* [13]. Though some scientists believed that the anionic residues in Melittin can neutralize the C‐terminal cationic Melittin, the precise mechanisms underlying the biocompatibility of Melittin to *A. mellifera* are inadequately explained. Furthermore, from a globally distributed 52,515 assembled metagenomes, a recent study has identified exceedingly higher numbers of AMP‐resistant genes in human‐involved environments (e.g., gut) than in other habitats [14]. The result indicates the existence of complex mutual adaptions between environmental microorganisms and AMP genes of human hosts.

In summary, AMPs exemplify the principle that “life finds a way,” showcasing the intricate relationship between evolutionary processes and biological function. The coevolution between AMP genes and particular microorganisms is a never‐ending story. AMPs are hypervariable macromolecules, especially in sequences and distributions; however, they are conservative and highly efficient weapons customized by universally selective pressures from microorganisms. Their evolutionary significance not only underscores their role as natural defense mechanisms but also provides valuable insights for the design of therapeutic agents. Harnessing the wisdom inherent in AMPs, along with advancements in computational methods, can facilitate the discovery and optimization of novel peptides tailored for clinical applications. Exploring the rich evolutionary tapestry of AMPs, scientists may unlock new avenues for combating antibiotic‐resistant microorganisms through naturally derived solutions.

## 2 BIOLOGICAL OMICS‐EMPOWERED COMPUTATIONAL APPROACH FINDS THE WAY

The advent of advanced computational techniques, such as supervised deep learning or generative models, has revolutionized the discovery and design of AMPs [6]. However, the concept of “Life finds a way” is not currently prioritized by computational scientists, as more efforts are devoted to developing elevated supervised or generative algorithms for AMP design. Only a few efforts have focused on mining proteomes and metagenomes. Compared with the previous approach, this commentary emphasizes the aspects of “nature's own evolutionary solutions” for developing novel AMPs.

One of the representative studies is the HydrAMP proposed for generating AMP sequences. HydrAMP contains two modes including unconstrained and analog generation [15]. Fundamental breakthroughs of HydrAMP lie in accurately identifying AMPs analogs and distinguishing between AMPs and non‐AMPs. However, it was designed for analog generation, and its performance in generating unconstrained peptides was merely evaluated via statistical analysis of physiochemical features, risky for designing novel AMPs dissimilar to the known AMPs. Furthermore, the Molecular Dynamic procedures in this work should be used carefully, as force field selections and simulation scales may lead to bias in predictions. A subsequent work focusing on generative models also revealed similar concerns [16]. An additional work in 2023 developed a coarse‐to‐fine pipeline covering billions of peptides with 6–9 amino acids in length for discovering potent AMPs. Though covering all possibilities, it seems like looking for a needle in a haystack, with unreasonable computational consumption, and low successful rates [17]. Such strategies would not be applicable to AMPs with higher length and high‐throughput tasks. In summary, all these studies overfocus on improving algorithms, without considering “life finds a way.”

Using biologically informative data undoubtedly increases the hit rates of medically useful AMPs with selective specificities. Furthermore, by integrating biological data, simple AI algorithms can effectively hit the targets with limited side effects. Using simple random forest classifiers, the identification of encrypted AMPs from human microbiome data hit multiple AMP candidates with low off‐target activities to gut commensals; In mouse models of skin abscesses and thigh infections, the leading AMP, prevotellin‐2, showed similar antibacterial efficacy to polymyxin B in vivo [18]. Furthermore, the random forest model made the “de‐extinction” of ancient AMPs from ancient Neanderthal paleoproteomes, with representatives showing efficacy against *Acinetobacter baumannii* in vivo [19]. A subsequent study in 2024 constructed the “AMP‐Sphere,” representing habitats‐specific AMP resources from global metagenomes; Approximately one million new AMPs were discovered from the “AMP‐Sphere” using simple machine learning, with leading peptides showing anti‐infection effects in vivo [20]. These studies pinpointed that the accurate identification of small open reading frames encoding AMP or antimicrobial fragments from larger proteins is a key step to improve success rates. Instead of using complicated algorithms, some studies merely used simple machine learning algorithms, indicating the importance of biological omics.

In summary, while diverse biological omics have undeniably accelerated the development of AMPs, the underlying evolutionary mechanisms that drive the formation of these sequences remain poorly understood. There is a pressing need for a more balanced approach that combines the strengths of advanced algorithms with insights drawn from biological evolution, rather than an overreliance on complex computational techniques. Insights inspired by these biological omics would enhance our understanding of how host AMPs distinguish pathogenic species and host cells, as well as the good biocompatibility and immune activity.

## 3 CONCLUSION

**Figure 1:**
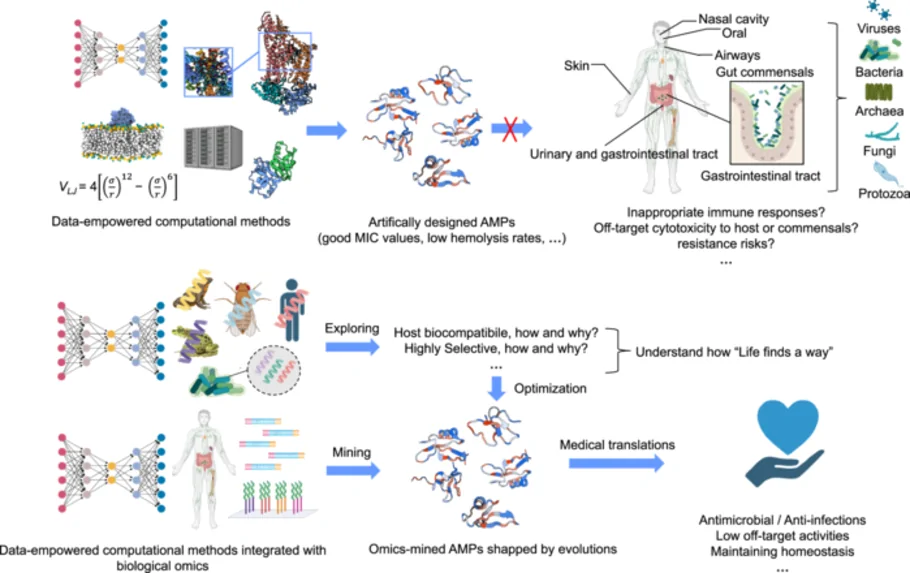
Summary of data‐empowered approach integrated with biological omics. The utilization of diverse omics data sets allows for a more nuanced analysis of organismal interactions and microbial resistance mechanisms, facilitating the identification of novel antimicrobial peptides (AMPs) with enhanced efficacy. By incorporating the hidden information of evolutionary biology perspectives, the framework potentially leverages the natural selection processes that have shaped the diversity of AMPs in different organisms, guiding the design of peptides with optimized properties. The holistic approach, combining computational algorithms with evolutionary biology views, holds the potential to revolutionize the way we identify and design AMPs, leading to more reliable and safe therapeutic candidates.

As antibiotic resistance continues to pose a dangerous threat to public health, the need for innovative solutions becomes increasingly urgent. AMPs represent a type of attractive macromolecules for developing antimicrobial drugs and their identification was accelerated by data‐empowered computational approaches. However, the risky concerns of inappropriate immune responses or low biocompatibility seem to be challenges to the current computational technologies. Alternatively, integrating the biological omics data (e.g., meta‐genomes, transcriptomes, proteomes, and other omics data) undoubtedly improves the success rates of hitting medically useful candidates with low cytotoxicity, low hemolysis rates, or inappropriate immune inferences. The concept of “life finds a way,” leveraging the evolutionary insights within these biological omics, represents a promising strategy. This holistic approach combines advanced algorithms with evolutionary biology, leading to more reliable and safe therapeutic candidates (Figure 1). The pursuit of safe and effective antimicrobial therapies through AMPs is a multifaceted challenge that requires collaboration across computational and biological disciplines. Overall, as Ian Malcolm said in “Jurassic Park,” “Life will not be contained. Life breaks freer. It expands to new territories and crashes through barriers painfully, maybe even dangerously, but, well, there it is.”

## AUTHOR CONTRIBUTIONS

**Sizhe Chen**: Conception and design, original and final manuscript writing and proofreading, data analysis and interpretation, figure presentation, table presentation, document collection. **Qi Su**: Manuscript revision, and final approval of the manuscript. All authors have read the final manuscript and approved it for publication.

## CONFLICT OF INTEREST STATEMENT

The authors declare no conflict of interest.

## ETHICS STATEMENT

No animals or humans were involved in this study.

## Supporting information

The online version contains supplementary figures and tables available.

**Figure S1.** Statistical summary of AMPs data from publicly available databases.

**Table S1.** Representative databases depositing data relevant to AMPs.
**Table S2.** Representative computational tools for exploiting AMPs in recent years.

## Data Availability

Data sharing is not applicable to this article as no data sets were generated or analyzed during the current study. No new data and scripts were used for this commentary. Supplementary information (graphical abstract, slides, videos, Chinese translated version, and update materials) may be found in the online DOI or iMeta Science http://www.imeta.science/imetaomics/.
