# Supplementary figures and images for: Insights into the identification of antimicrobial peptides: A multidisciplinary observation
Source: IMetaOmics. 2024 Nov 5;1(2):e41. doi: 10.1002/imo2.41 (PMC12806194; doi:10.1002/imo2.41)

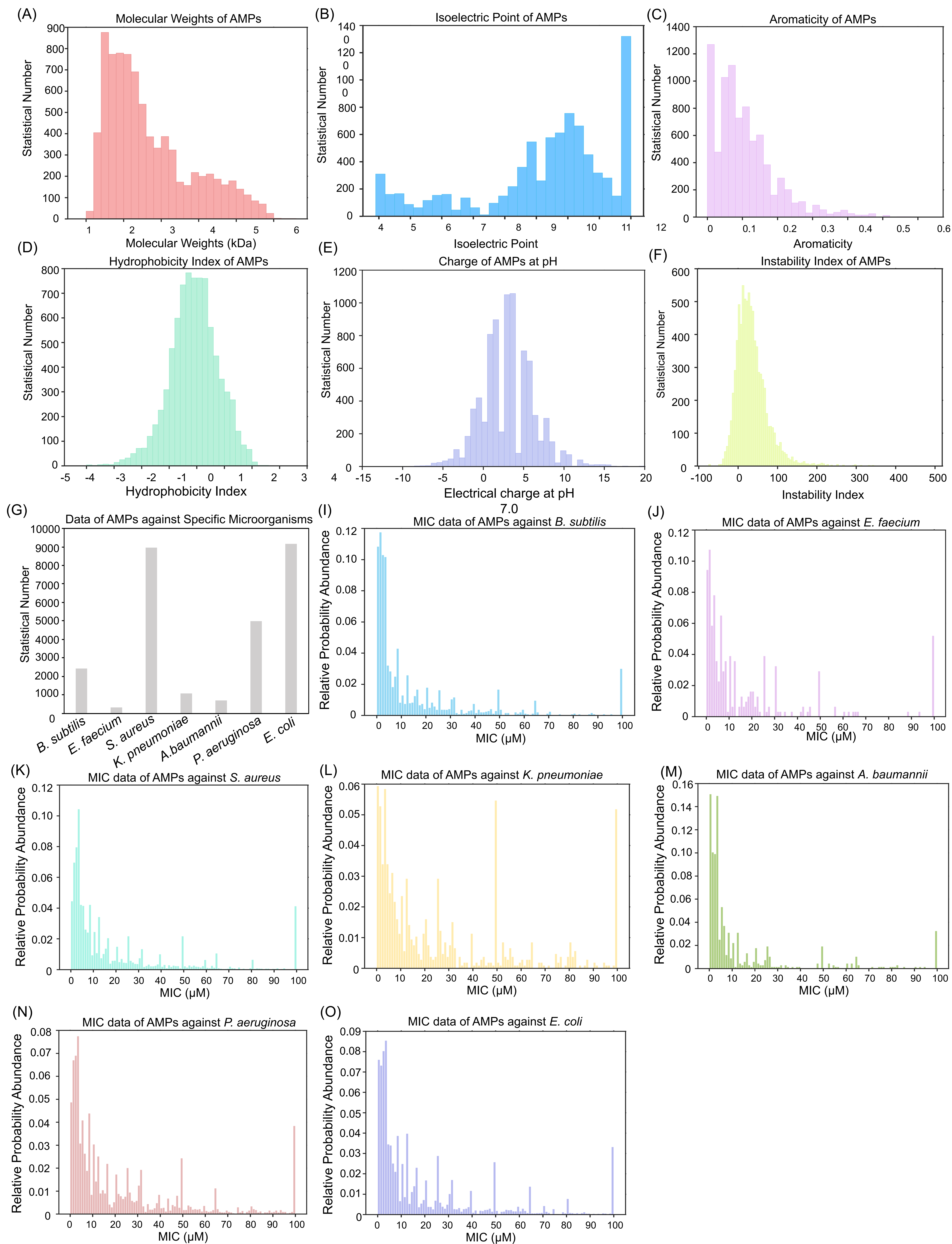

Supplement: Supplementary file 1 — Figure S1. Statistical summary of AMPs data from publicly available databases. [file IMO2-1-e41-s001.pdf]
