# Supplementary material for: Insights into the identification of antimicrobial peptides: A multidisciplinary observation
Source: IMetaOmics. 2024 Nov 5;1(2):e41. doi: 10.1002/imo2.41 (PMC12806194; doi:10.1002/imo2.41)
Supplement: Supplementary file 2 — Table S1. Representative databases depositing data relevant to AMPs. Table S2. Representative computational tools for exploiting AMPs in recent years. [file IMO2-1-e41-s002.docx]

**Supporting Information to**

**Insights into the identification of antimicrobial peptides: a multidisciplinary observation**

**Running Title:** Harnessing evolutionary insights behind multi-omics for exploiting AMPs

Sizhe Chen^1,2^, Qi Su^1,2*^

^1^ Microbiota I-Center (MagIC), Hong Kong SAR, China

^2^ Department of Medicine and Therapeutics, Faculty of Medicine, The Chinese University of Hong Kong, Hong Kong Special Administrative Region, Hong Kong, China

^*^Corresponding authors: qisu@cuhk.edu.hk (Qi Su)

**
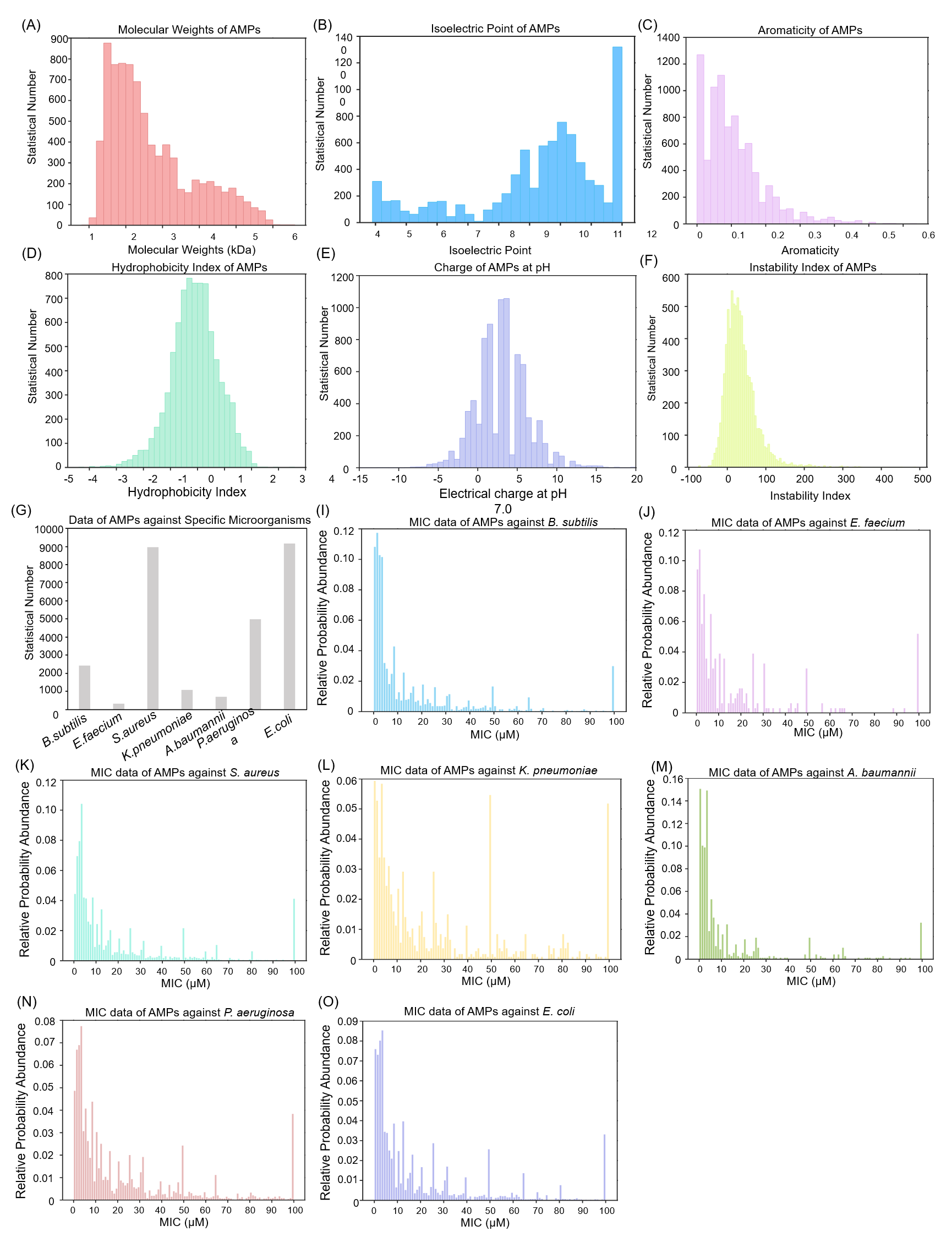
Figure S1 Statistical summary of AMPs data from publicly available databases.** The statistical features of AMPs with characteristics of (A) molecular weights (B) Isoelectric Point, (C) Aromaticity, (D) Hydrophobicity Index, (E) Charge at pH 7.0, (F) Instability Index summarized from four publicly available (ADAM [1], CAMP [2], APD [3], and LAMP [4]). (G) Deposited experimental data tags against *Bacillus subtilis*, *Enterococcus faecium*, *Staphylococcus aureus*, *Klebsiella pneumoniae*, *Acinetobacter baumannii*, *Pseudomonas aeruginosa*, *Escherichia coli* summarized from database of GRAMPA [5]. The exact experimental MIC values (< 100 *μ*M) collected from GRAMPA library for (H) *B. subtilis*, (I) *E. faecium*, (J) *E.coli*, (K) *S. aureus*, (L) *K. pneumoniae*, (M) *A. baumannii*, and (n) *P. aeruginosa* are statistically summarized.

**Table S1.** Representative databases depositing data relevant to AMPs

| **Database** | **Sequence** | **Experimental Structure** | **Link** | **Reference** |
| --- | --- | --- | --- | --- |
| CAMP | 24,243 | 933 | <http://camp.bicnirrh.res.in/> | [2] |
|  |  |  |  |  |
| APD | 3,940 | 486 | <https://aps.unmc.edu/AP/> | [3] |
|  |  |  |  |  |
| DBAASP | 22,119 | 619 | <https://dbaasp.org/home> | [6] |
|  |  |  |  |  |
| DRAMP | 29,948 | 136 | <http://dramp.cpu-bioinfor.org/> | [7] |
|  |  |  |  |  |
| DADP | 2,571 | NA | <http://split4.pmfst.hr/dadp/> | [8] |
|  |  |  |  |  |
| AMPDB v1 | 59,122 | NA | <https://bblserver.org.in/ampdb/ampdb-home> | [9] |
|  |  |  |  |  |
| YADAMP | 2,525 | NA | <http://www.yadamp.unisa.it/> | [10] |
|  |  |  |  |  |
| LAMP | 23,253 | NA | <http://biotechlab.fudan.edu.cn/database/lamp> | [4] |
|  |  |  |  |  |
| dbAMP | 33,065 | ~30,000 | <https://awi.cuhk.edu.cn/dbAMP/index.php> | [11] |
|  |  |  |  |  |
| ANTIPSEUDOBASE | 560 | NA | <http://bims.pasteur.tn:3838/APP/> | [12] |
|  |  |  |  |  |
| ADAPTABLE | NA | NA | <https://www.hammamilab.org/resources/BACTIBASE> | [13] |
|  |  |  |  |  |
| InverPEP | 702 | NA | <http://ciencias.medellin.unal.edu.co/gruposdeinvestigacion/prospeccionydisenobiomoleculas/InverPep/public/home_en> | [14] |
|  |  |  |  |  |
| ADAM | 7007 | 759 | <http://bioinformatics.cs.ntou.edu.tw/ADAM/index.html> | [3] |
|  |  |  |  |  |
| BaAMPs | 221 | NA | <http://www.baamps.it/> | [15] |
|  |  |  |  |  |
| AntiTbPdb | 1010 | NA | <https://webs.iiitd.edu.in/raghava/antitbpdb/index.html> | [16] |
|  |  |  |  |  |

**Table S2.** Representative computational tools for exploiting AMPs in recent years

| **Name** | **Methods** | **Web server or original codes** | **Experimental Validation** | **Reference** |
| --- | --- | --- | --- | --- |
| PGAT-ABPp | Graph attention network based on a pretrained protein language model | <https://github.com/moonseter/PGAT-ABPp/> | No | [17] |
| AMPidentifier | Densely-connected convolutional network with attention module | <https://github.com/ChenSizhe13893461199/Fast-AMPs-Discovery-Projects> | Yes | [18] |
| HydrAMP | Conditional variational autoencoder leverages parameter-controlled creativity | <https://github.com/szczurek-lab/hydramp> | Yes | [19] |
| deepAMP | Peptide language-based deep generative framework | <https://github.com/jimmyrate/deepAMP> | Yes | [20] |
| Generative AMP model | A method combines attribute-controlled deep generative models and physics-driven simulations | <https://github.com/IBM/controlled-peptide-generation> | Yes | [21] |
| Multi-NLP model | A multiple natural language processing model including frameworks of LSTM, Attention and BERT | <https://github.com/mayuefine/c_AMPs-prediction> | Yes | [22] |
|  |  |  |  |  |
| AMP-RNNpro | RNN model designed with eight feature encoding methods | <http://13.126.159.30/> | No | [23] |
| APEX model | A multitask deep learning strategy for mining proteomes of extinct organisms | <https://gitlab.com/machine-biology-group-public/apex> | Yes | [24] |
| DMAMP | A multi-task model utilizing convolutional neural network, residual blocks, and two fully connected layers to simultaneously predict AMPs and their activities | <https://github.com/unimqz/DMAMP> | No | [25] |
|  |  |  |  |  |
| Multi-CGAN | A deep generative model that can generate AMP sequences with diverse desired attributes | <https://github.com/hqyu/Multi-CGAN> | No | [26] |
|  |  |  |  |  |
| TransImbAMP | A deep learning model using the transformer architecture and natural language processing | <https://github.com/BiOmicsLab/TransImbAMP> | No | [27] |
| MoFormer | A novel model designed by using conditional Transformer joint multi-modal fusion descriptor | NA | No | [28] |
| panCleave | Simple computational model combined with protein cleavage predictor strategy | <https://gitlab.com/machine-biology-group-public/pancleave> | Yes | [29] |
| AMPGAN | A delicately designed bidirectional conditional generative adversarial model | <https://gitlab.com/vail-uvm/amp-gan> | No | [30] |
| PepVAE | A peptide generation model based on variational autoencoder and activity prediction models | NA | No | [31] |

**References:**

1. Lee, Hao-Ting, Chen-Che Lee, Je-Ruei Yang, Jim Z C Lai, Kuan Y Chang. 2015. “A large-scale structural classification of antimicrobial peptides.” *BioMed Research International* 2015: 475062. <https://doi.org/10.1155/2015/475062>

2. Gawde, Ulka, Shuvechha Chakraborty, Faiza Hanif Waghu, Ram Shankar Barai, Ashlesha Khanderkar, Rishikesh Indraguru, Tanmay Shirsat, Susan Idicula-Thomas. 2023. “CAMPR4: a database of natural and synthetic antimicrobial peptides.” *Nucleic Acids Research* 51: D377-D383. <https://doi.org/10.1093/nar/gkac933>

3. Wang, Guangshun, Xia Li, Zhe Wang. 2016. “APD3: the antimicrobial peptide database as a tool for research and education.” *Nucleic Acids Research* 44: D1087-D1093. <https://doi.org/10.1093/nar/gkv1278>

4. Zhao, Xiaowei, Hongyu Wu, Hairong Lu, Guodong Li, Qingshan Huang. 2013. “LAMP: A Database Linking Antimicrobial Peptides.” *Plos One* 8: e66557. <https://doi.org/10.1371/journal.pone.0066557>

5. Witten, Jacob, Zack Witten. 2019. “Deep learning regression model for antimicrobial peptide design.” *bioRxiv* 32: 692681. <https://doi.org/10.1101/692681>

6. Pirtskhalava, Malak, Anthony A Amstrong, Maia Grigolava, Mindia Chubinidze, Evgenia Alimbarashvili, Boris Vishnepolsky, Andrei Gabrielian, Alex Rosenthal, Darrell E Hurt, Michael Tartakovsky. 2021. “DBAASP v3: database of antimicrobial/cytotoxic activity and structure of peptides as a resource for development of new therapeutics.” *Nucleic Acids Research* 49: D288–D297. <https://doi.org/10.1093/nar/gkaa991>

7. Kang, Xinyue, Fanyi Dong, Cheng Shi, Shicai Liu, Jian Sun, Jiaxin Chen, Haiqi Li, Hanmei Xu, Xingzhen Lao, Heng Zheng. 2019. “DRAMP 2.0, an updated data repository of antimicrobial peptides.” *Scientific Data*, 6: 148. <https://doi.org/10.1038/s41597-019-0154-y>

8. Novković, Mario, Juraj Simunić, Viktor Bojović, Alessandro Tossi, Davor Juretić. 201.2 “DADP: the database of anuran defense peptides.” *Bioinformatics*, 28: 1406-1407. <https://doi.org/10.1093/bioinformatics/bts141>

9. Mondal, Rajat Kumar, Debarup Sen, AnkishArya, Sintu Kumar Samanta. 2023. “Developing anti-microbial peptide database version 1 to provide comprehensive and exhaustive resource of manually curated AMPs.” *Scientific Reports*, 13: 17843. <https://doi.org/10.1038/s41598-023-45016-3>

10. Piotto, Stefano P, Lucia Sessa, Simona Concilio, Pio Iannelli. 2021. “YADAMP: yet another database of antimicrobial peptides.” *International Journal of Antimicrobial Agents*, 39(4): 346-351. <https://doi.org/10.1016/j.ijantimicag.2011.12.003>

11. Jhong, Jhih Hua, Lantian Yao, Yuxuan Pang, Zhongyan Li, Chia Ru Chung, Rulan Wang, Shangfu Li. 2022. “dbAMP 2.0: updated resource for antimicrobial peptides with an enhanced scanning method for genomic and proteomic data.” *Nucleic Acids Research*, 50(D1): D460-D470. <https://doi.org/10.1093/nar/gkab1080>

12. Zouhir, Abdelmajid, Oussama Souiai, Emna Harigua, Ammar Cherif, Aymen Ben Chaalia, Khaled Sebei. 2023. “ANTIPSEUDOBASE: Database of Antimicrobial Peptides and Essential Oils Against *Pseudomonas*.” *International Journal of Peptide Research and Therapeutics*, 29: 37. <https://doi.org/10.1007/s10989-023-10511-8>

13. Ramos-Martín Francisco, Thibault Annaval, Sébastien Buchoux, Catherine Sarazin, Nicola D'Amelio. 2019. “ADAPTABLE: a comprehensive web platform of antimicrobial peptides tailored to the user's research.” Life science alliance, 2 (6): e201900512. <https://doi.org/10.26508/lsa.201900512>

14. Gómez, Esteban A, Paula Giraldo, Sergio Orduz. 2017. “InverPep: A database of invertebrate antimicrobial peptides.” *Journal of Global Antimicrobial Resistance*, 8: 13-17. <https://doi.org/10.1016/j.jgar.2016.10.003>

15. Luca, Mariagrazia Di, Giuseppe Maccari, Giuseppantonio Maisetta, Giovanna Batoni. 2015. “BaAMPs: the database of biofilm-active antimicrobial peptides.” *Biofouling*, 31 (2): 193-9. <https://doi.org/10.1080/08927014.2015.1021340>

16. Usmani, Salman Sadullah, Rajesh Kumar, Vinod Kumar, Sandeep Singh, Gajendra P S Raghava. 2018. “AntiTbPdb: a knowledgebase of anti-tubercular peptides.” *Database (Oxford)*, 2018: bay025. <https://doi.org/10.1093/database/bay025>

17. Hao, Yuelei, Xuyang Liu, Haohao Fu, Xueguang Shao, Wensheng Cai. 2024. “PGAT-ABPp: harnessing protein language models and graph attention networks for antibacterial peptide identification with remarkable accuracy.” *Bioinformatics*, 40 (8): btae497. <https://doi.org/10.1093/bioinformatics/btae497>

18. Chen, Sizhe, Huitang Qi, Xingzhuo Zhu, Tianxiang Liu, Yutin Fan, Qiuyu Gong, Cangzhi Jia, Tian Liu. 2024. “The discovery of antimicrobial peptides from the gut microbiome of cockroach *Blattella germanica* using deep learning pipeline.” *bioRxiv*. <https://doi.org/10.1101/2024.02.12.580024>

19. Szymczak, Paulina, Marcin Możejko, Tomasz Grzegorzek, Radosław Jurczak, Marta Bauer, Damian Neubauer, Karol Sikora. 2023. “Discovering highly potent antimicrobial peptides with deep generative model HydrAMP.” *Nature Communication*, 14: 1453. <https://doi.org/10.1038/s41467-023-36994-z>

20. Li, Tingting, Xuanbai Ren, Xiaoli Luo, Zhuole Wang, Zhenlu Li, Xiaoyan Luo, Jun Shen, Yun Li, Dan Yuan, Ruth Nussinov, Xiangxiang Zeng, Junfeng Shi, Feixiong Cheng. 2024. “A Foundation Model Identifies Broad-Spectrum Antimicrobial Peptides against Drug-Resistant Bacterial Infection.” *Nature Communications*, 15: 7538. <https://doi.org/10.1038/s41467-024-51933-2>

21. Das, Payel, Tom Sercu, Kahini Wadhawan, Inkit Padhi, Sebastian Gehrmann, Flaviu Cipcigan, Vijil Chenthamarakshan. 2021. “Accelerated antimicrobial discovery via deep generative models and molecular dynamics simulations.” *Nature Biomedical Engineering*, 5: 613–623. <https://doi.org/10.1038/s41551-021-00689-x>

22. Ma, Yue, Zhengyan Guo, Binbin Xia, Yuwei Zhang, Xiaolin Liu, Ying Yu, Na Tang, Xiaomei Tong, Min Wang, Xin Ye, Jie Feng, Yihua Chen, Jun Wang. 2022. “Identification of antimicrobial peptides from the human gut microbiome using deep learning.” Nature Biotechnology, 40: 921–931. <https://doi.org/10.1038/s41587-022-01226-0>

23. Shaon, Md. Shazzad Hossain, Tasmin Karim, Md. Fahim Sultan, Md. Mamun Ali, Kawsar Ahmed, Md. Zahid Hasan, Ahmed Moustafa, Francis M. Bui, Fahad Ahmed Al-Zahrani. 2024. “AMP-RNNpro: a two-stage approach for identification of antimicrobials using probabilistic features.” Scientific Reports, 14: 12892. <https://doi.org/10.1038/s41598-024-63461-6>

24. Wan, Fangping, Marcelo D. T. Torres, Jacqueline Peng, Cesar de la Fuente-Nunez et al. 2024. “Deep-learning-enabled antibiotic discovery through molecular de-extinction.” *Nature Biomedical Engineering*, 8: 854–871. <https://doi.org/10.1038/s41551-024-01201-x>

25. Meng, Qiaozhen, Genlang Chen, Shixin Zheng, Yulai Lin, Bin Liu, Jijun Tang. 2024. "DMAMP: A deep-learning model for detecting antimicrobial peptides and their multi-activities." *IEEE/ACM Transactions on Computational Biology and Bioinformatics*, 1-10 <https://doi.org/10.1109/TCBB.2024.3439541>

26. Yu, Haoqing, Ruheng Wang, Jianbo Qiao, Leyi Wei. 2023. “Multi-CGAN: Deep Generative Model-Based Multiproperty Antimicrobial Peptide Design.” *Journal of Chemical Information and Modeling*, 64: 1. <https://doi.org/10.1021/acs.jcim.3c01881>

27. Pang, Yuxuan, Lantian Yao, Jingyi Xu, Zhuo Wang, Tzong-Yi Lee. 2022. “Integrating transformer and imbalanced multi-label learning to identify antimicrobial peptides and their functional activities.” *Bioinformatics*, 38: 24. <https://doi.org/10.1093/bioinformatics/btac711>

28. Wang, Li, Xiangzheng Fu, Jiahao Yang, Xinyi Zhang, Xiucai Ye, Yiping Liu, Tetsuya Sakurai, Xiangxiang Zeng. 2024. “MoFormer: Multi-objective Antimicrobial Peptide Generation Based on Conditional Transformer Joint Multi-modal Fusion Descriptor.” *arXiv*. <https://doi.org/10.48550/arXiv.2406.02610>

29. Maasch, Jacqueline R.M.A., Marcelo D.T. Torres, Marcelo C.R. Melo, Cesar de la Fuente-Nunez. 2023. “Molecular de-extinction of ancient antimicrobial peptides enabled by machine learning.” *Cell Host & Microbe*, 31(8): 1260-1274.e6. <https://doi.org/10.1016/j.chom.2023.07.001>

30. Oort, Colin M Van, Jonathon B Ferrell, Jacob M Remington, Safwan Wshah, Jianing Li. 2022. “AMPGAN v2: Machine Learning-Guided Design of Antimicrobial Peptides.” *Journal of Chemical Information and Modeling*. 61(5): 2198-2207. <https://doi.org/10.1021/acs.jcim.0c01441>

31. Dean, Scott N., Scott N. Dean, Jerome Anthony E. Alvarez, Dan Zabetakis, Scott A. Walper, Anthony P. Malanoski. 2021. “PepVAE: Variational Autoencoder Framework for Antimicrobial Peptide Generation and Activity Prediction.” Frontiers in Microbiology, 12: 725727. <https://doi.org/10.3389/fmicb.2021.725727>
